# How RA Associated HLA-DR Molecules Contribute to the Development of Antibodies to Citrullinated Proteins: The Hapten Carrier Model

**DOI:** 10.3389/fimmu.2022.930112

**Published:** 2022-06-10

**Authors:** Jean Roudier, Nathalie Balandraud, Isabelle Auger

**Affiliations:** ^1^ Faculté de Médecine, Aix Marseille Université, Marseille, France; ^2^ Assistance Publique Hôpitaux de Marseille, Marseille, France; ^3^ INSERM U1097 Immunogénétique de la Polyarthrite Rhumatoïde, Marseille, France; ^4^ Faculté des Sciences, Aix Marseille Université, Marseille, France

**Keywords:** rheumatoid arthritis, HLA-DR, ACPA, pad4, hapten carrier, tolerization

## Abstract

The risk to develop ACPA positive rheumatoid arthritis (RA), the most destructive type of autoimmune arthritis, is carried by HLA-DRB1 alleles containing a 5 amino acid motif: the shared epitope (SE). RA is preceded by the emergence of disease specific anti citrullinated protein antibodies (ACPA). SE positive HLA-DRB1 alleles are associated with ACPA and ACPA positive RA, not with ACPA negative RA, suggesting that ACPA contribute to the pathogenesis of RA. Understanding how HLA-DRB1 genotypes influence ACPA could lead to a curative or preventive treatment of RA. The “Shared epitope binds citrullinated peptides “ hypothesis suggests that RA associated HLA-DR alleles present citrullinated peptides to T cells that help ACPA producing B cells. The “Hapten carrier model” suggests that PAD4 is the target of the T cells which help ACPA specific B cells through a hapten carrier mechanism in which PAD4 is the carrier and citrullinated peptides are the haptens. Direct binding assay of citrullinated peptides to purified HLA-DR molecules does not support the “shared epitope binds citrullinated peptides” hypothesis. The Odds Ratios to develop ACPA positive RA associated with each of 12 common HLA-DRB1 genotypes match the probability that the two HLA-DR molecules they encode can bind at least one peptide from PAD4, not from citrullinated fibrinogen. Thus, PAD4 tolerization might stop the carrier effect and switch off production of ACPA.

## Rheumatoid Arthritis: From HLA-DR to ACPA

Rheumatoid arthritis (RA) stands out as the most destructive, best characterized type of autoimmune arthritis. In its most classical “ACPA positive” form which is the object of this study, it develops on a genetic background dominated by HLA-DRB1 genotypes (the two HLA-DRB1 genes inherited by every individual) and it is preceded by the development of particular autoantibodies, ACPA (anti citrullinated protein antibodies), that recognize citrullin residues on many different proteins.

ACPA are likely to cause RA and therefore, the mechanism by which they develop is central to understanding RA and figuring a way to curing or preventing it.

This review is an attempt at explaining how HLA-DRB1 genes influence the development of ACPA in the classical ACPA positive subset of RA.

## Association Between HLA-DRB1 and Classical ACPA Positive RA

Two thirds of patients with RA express particular HLA-DR molecules that share a 5 aminoacid motif on their B1 chain. This motif, the “shared epitope”, is charged and basic. Common HLA-DRB1 alleles encoding the shared epitope are HLA-DRB1*04:01,*04:04, *0405, (HLA-DR4), HLA-DRB1*01:01 (HLA-DR1), HLA-DRB1*10:01 (HLA-DR10) (1).

There is a dose effect of shared epitope positive HLA-DRB1 alleles on the risk to develop RA: subjects with two shared epitope positive HLA-DRB1 alleles are at higher risk than those with one shared epitope positive HLA-DRB1 allele, and subjects with no shared epitope positive HLA-DRB1 allele are at the lowest risk. For most HLA-DRB1 genotypes, OR to develop RA range from 28 to 0.2 (2).

## Association Between HLA-DR and ACPA

Because HLA-DR molecules contribute to the development of antibody responses by presenting antigenic peptides to helper T lymphocytes, it was anticipated that RA associated HLA-DR molecules would be associated with ACPA.

Indeed, this was confirmed using “anti CCP” (anti Cyclic Citrullinated Peptides) or “anti citrullinated fibrinogen” testing to detect ACPA (3, 4).

The association of shared epitope encoding HLA-DRB1 alleles and RA did not hold for ACPA negative RA, suggesting that HLA-DRB1 genes contribute to RA by allowing the development of ACPA (5).

## Do SE Positive HLA-DR Molecules Bind Citrullinated Peptides Better?

In a seminal article published in 2003, Jonathan Hill and Eva Cairns showed that a citrullinated peptide from Vimentin, Vim65-77, could elicit T cell responses in HLA-DRB1*0401 transgenic mice, under its citrullinated form, Vim R70Cit, but not under its native, arginine form, Vim 65-77 (6). In collaboration with the Sette group, Hill and Cairns showed that Vim R70 Cit bound RA associated HLA-DRB1*04:01, *04:04 and *01:01 with higher affinity than Vim65-77. From this observation based on the binding properties of one peptide from vimentin and its one citrullinated variant to 8 different HLA-DR molecules, came the hypothesis that shared epitope positive HLA-DR molecules bound citrullinated peptides better than shared epitope negative HLA-DR molecules, thus explaining the RA, ACPA, HLA-DR association (6).

At the same time, while analyzing the association of anti citrullinated fibrinogen antibodies with shared epitope positive HLA-DR molecules, we studied the binding of 167 peptides covering the entire alpha and beta chains of fibrinogen under their native and citrullinated forms to 5 different HLA-DR molecules. We found no evidence of preferential binding of citrullinated fibrinogen peptides to shared epitope positive HLA-DR molecules (4).

In 2013, Scally and al performed cristallographic studies of the binding of VimR70C to HLA-DRB1*04:01, HLA-DRB1*04:04, HLA-DRB1*04:02. They showed direct binding of Vim R70Cit to HLA- DRB1*04:01, *04:04 and HLA-DRB1*04:02, the latter unexpected, because HLA-DRB1*04:02 does not contain the shared epitope and is not associated with RA (7).

Finally, in 2017, the Sette group revisited their original Vim65-77 HLA-DR binding studies, this time using 200 native and citrullinated peptides from Vimentin and testing their binding to 28 HLA-DR alleles. They concluded that “RA associated epitopes in their wild type and citrullinated forms have variable binding patterns to HLA class II alleles and a consistent impact of citrullination is not apparent” (8).

Thus, the “shared epitope binds citrullinated peptides” hypothesis is not based on a general property of citrullinated peptides. Even if a few citrullinated epitopes may be relevant to the triggering of ACPA, we believe that another explanation of the RA/ACPA/HLA-DR association has to be found.

## RA Patients With No ACPA: “Anti PAD4” Antibodies

In the 2000s, ACPA testing became part of the diagnosis of RA and we wondered whether, in the roughly one third of our RA patients without ACPAs, we could identify other antigen/antibody systems that could prove helpful for diagnosis.

We used 8000 human protein arrays from Invitrogen to screen the sera of patients with chronic inflammatory arthritis (9). Unexpectedly, one antigen stood out as highly associated with RA, the citrullinating enzyme PAD4. When tested in early RA or in ACPA negative RA, the association was even stronger (10, 11).

PAD4 had already been described as an autoantigen in RA (12). Moreover, antibodies to PAD4 had been shown to develop before the onset of RA (13).

This lead us propose that “anti–citrullinated protein” immunization could arise because many proteins being citrullinated by PAD4 could become the target of autoantibody responses helped by T cell recognition of PAD4 in the protein complex formed with PAD4 (10). This is the “hapten carrier model”.

## PAD Proteins as Immunological Carriers: The Hapten Carrier Model

The finding of “anti PAD4 IgG antibodies” in the sera of patients with RA implied the existence of helper T lymphocytes that recognized peptides from PAD4.

**Figure 1:**
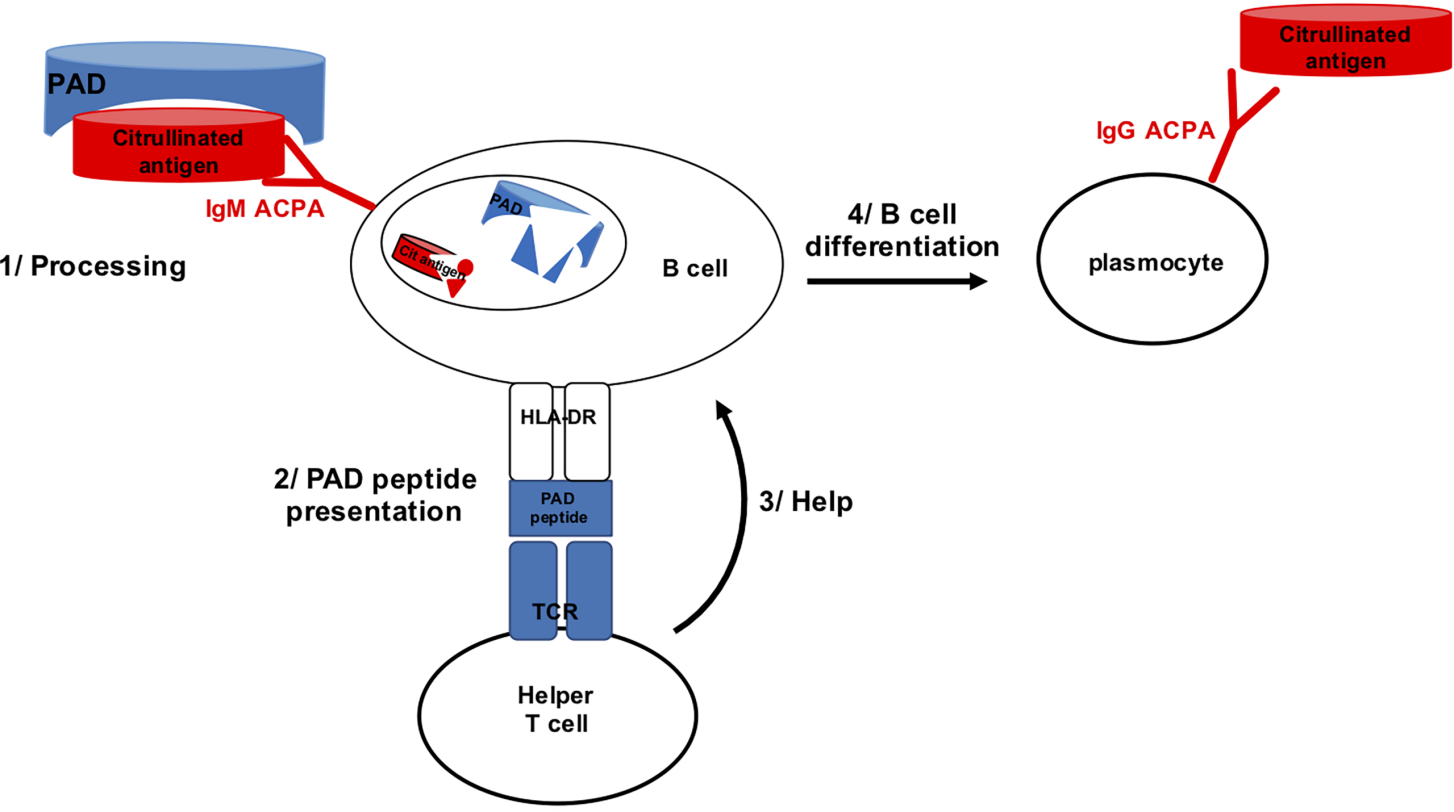
B cell specific for citrullinated epitope on PAD bound peptide can process PAD and present PAD peptide to PAD specific helper T cell.

Because PAD4 binds many different proteins for citrullination, it was conceivable that B lymphocytes specific for citrullinated epitopes might recognize them during citrullination, when bound by PAD4. Thus, citrullinated epitope specific B lymphocytes might bind citrullinated epitopes complexed with PAD4, process the PAD4/citrullinated peptide complex and present a peptide from PAD4 processed from the complex to helper T cells. Then, B cells specific for citrullinated epitopes might benefit from the help of PAD4 specific helper T cells (**Figure 1**).

To test this hypothesis, we immunized normal, non-autoimmune mice with human or murine PAD4 or PAD2. We observed the development of anti citrullinated fibrinogen peptide antibodies in 20% of these mice with an influence of the MHC background on the frequency of the ACPA response. The best responders were C3H mice whose IE beta chain (the murine equivalent of HLA-DRB1) is almost identical with HLA-DRB1*0401 in the shared epitope region (14).

In patients with joint disease, we found that approximately half of patients with RA have antibodies to PAD4. More important, half of the patients with RA have T cell proliferative responses to PAD4 and this is unusual because immunosuppressive treatments impair detection of T cell responses. Finally, having both antibodies and T cells specific for PAD4 was specific of RA (15).

## PAD4 Immunization in Different Mouse Strains: Confirmation of the Hapten Carrier Model and Development of an ACPA Monitoring Array in the Mouse

Immunizing different mouse strains with human PAD4 [C3H (H2k), C57BL6 (H2b), BalbC (H2d), DBA2 (H2d)], allowed us identify high responders, like C3H and C57BL6 and low responders like BalbC and DBA2 and to develop a 169 peptide array that allows us monitor PAD4 induced ACPAs with great precision (16).

This model is now ready for analyzing the effect of PAD4 tolerization on the PAD4 induced ACPA response in the mouse.

## Which Model Explains the Data Better: “Shared Epitope Binds Citrullinated Peptides” or “Shared Epitope Binds Peptides From PAD4”?

In 2013, we had developed a table indicating the risk to develop ACPA positive RA for 106 of 136 HLA-DR genotypes, calculated on 3000 subjects (2).

We reasoned that the risk associated with each genotype should correlate with its binding properties vis à vis a relevant RA triggering antigen.

Thus, to compare the prediction of the two models, we calculated the probability for the two HLA-DR molecules encoded by each of 12 HLA-DRB1 genotypes to bind at least one peptide from PAD4 or native or citrullinated fibrinogen.

This was done by first testing binding of overlapping peptides from PAD4 or Fibrinogen to 5 different HLA-DR alleles. From the percentage of peptides bound by each allele, we evaluated the probability for a given pair of HLA-DR molecules to bind at least one peptide from PAD4, Fibrinogen or citrullinated Fibrinogen.

We found a strong correlation between PAD4 peptide binding and HLA-DRB1 genotypic risk for RA. By the same analysis, Fibrinogen or citrullinated Fibrinogen peptide binding did not correlate with HLA-DRB1 genotypic risk to develop RA (17).

In short, modelling of HLA-DR/peptide binding properties favors the”hapten carrier” model over the “shared epitope binds citrullinated peptides” hypothesis. Still, modelling is no definitive proof. Both models might coexist.

## Two Possible Models With Very Different Prospects

If ACPAs can trigger and drive classical ACPA positive RA, thus, understanding why they develop may be the clue to treating or preventing RA.

Two possible models for the development of ACPA exist: “the shared epitope bind citrullinated peptides” hypothesis and the “hapten carrier” model.

Even if these two models are not exclusive, the prospects for the development of a radical treatment of RA are very different under the light of each of these two models.

If the “Shared epitope binds citrullinated peptides” hypothesis is correct, then, thousands of helper T cell clones specific for thousands of citrullinated peptides and helping thousands of B lymphocyte clones may exist and be very difficult to inactivate.

Conversely, if, as we demonstrated, the “Hapten Carrier model” is correct, then, tolerization to PAD4 might be a direct way to switch off ACPAs…

## Author Contributions

JR: designed research, analysed data, wrote article IA: designed research, performed experiments, analysed data, reviewed article NB: performed experiments, analysed data, reviewed article. All authors contributed to the article and approved the submitted version.

## Funding

INSERM, Arthritis Fondation Clarins, Société Française de rhumatologie.

## Conflict of Interest

The authors declare that the research was conducted in the absence of any commercial or financial relationships that could be construed as a potential conflict of interest.

## Publisher’s Note

All claims expressed in this article are solely those of the authors and do not necessarily represent those of their affiliated organizations, or those of the publisher, the editors and the reviewers. Any product that may be evaluated in this article, or claim that may be made by its manufacturer, is not guaranteed or endorsed by the publisher.
